# Network Pharmacology and Molecular Docking Suggest the Mechanism for Biological Activity of Rosmarinic Acid

**DOI:** 10.1155/2021/5190808

**Published:** 2021-04-11

**Authors:** Minglong Guan, Lan Guo, Hengli Ma, Huimei Wu, Xiaoyun Fan

**Affiliations:** ^1^Department of Geriatric Respiratory and Critical Care, The First Affiliated Hospital of Anhui Medical University, Jixi Road 218, Hefei, Anhui 230022, China; ^2^Anhui Geriatric Institute, Jixi Road 218, Hefei, Anhui 230022, China; ^3^Anhui Key Laboratory of Geriatric Molecular Medicine, Anhui Medical University, Jixi Road 218, Hefei, Anhui 230022, China; ^4^Department of Biochemistry and Molecular Biology, School of Basic Medical Sciences, Anhui Medical University, Meishan Road 81, Hefei, Anhui 230032, China

## Abstract

Rosmarinic acid (RosA) is a natural phenolic acid compound, which is mainly extracted from Labiatae and *Arnebia*. At present, there is no systematic analysis of its mechanism. Therefore, we used the method of network pharmacology to analyze the mechanism of RosA. In our study, PubChem database was used to search for the chemical formula and the Chemical Abstracts Service (CAS) number of RosA. Then, the Traditional Chinese Medicine Systems Pharmacology Database and Analysis Platform (TCMSP) was used to evaluate the pharmacodynamics of RosA, and the Comparative Toxicogenomics Database (CTD) was used to identify the potential target genes of RosA. In addition, the Gene Ontology (GO) enrichment analysis and Kyoto Encyclopedia of Genes and Genomes (KEGG) pathway enrichment analysis of target genes were carried out by using the web-based gene set analysis toolkit (WebGestalt). At the same time, we uploaded the targets to the STRING database to obtain the protein interaction network. Then, we carried out a molecular docking about targets and RosA. Finally, we used Cytoscape to establish a visual protein-protein interaction network and drug-target-pathway network and analyze these networks. Our data showed that RosA has good biological activity and drug utilization. There are 55 target genes that have been identified. Then, the bioinformatics analysis and network analysis found that these target genes are closely related to inflammatory response, tumor occurrence and development, and other biological processes. These results demonstrated that RosA can act on a variety of proteins and pathways to form a systematic pharmacological network, which has good value in drug development and utilization.

## 1. Introduction

Traditional Chinese medicine and natural compounds contain a large number of active components. This provides more possibilities and opportunities for the development and the use of drugs. Rosmarinic acid (RosA) is one of the water-soluble natural phenolic acid compounds [1]. It is widely found in Labiatae and *Arnebia* plants such as *Rosmarinus officinalis*, *Melissa officinalis*, and *Hyssopus cuspidatus* Boriss [2]. Increasing studies have shown that RosA not only has strong anti-inflammatory activity but also has antibacterial, antivirus, and antitumor activity [3–7]. Moreover, RosA showed antiallergic inflammation, antifibrosis, and liver-protective effects [8–10]. So RosA has been paid more attention in the fields of pharmacy, food, cosmetics, and so on. However, the molecular mechanism induced by RosA and the corresponding cell phenotypic changes have not been systematically studied. At the same time, the use of computational methods to identify and predict drug targets and potential mechanisms is becoming a major method [11, 12]. This approach cannot only speed up the process of drug discovery and design but also save money, time, and energy.

**Figure 1:**
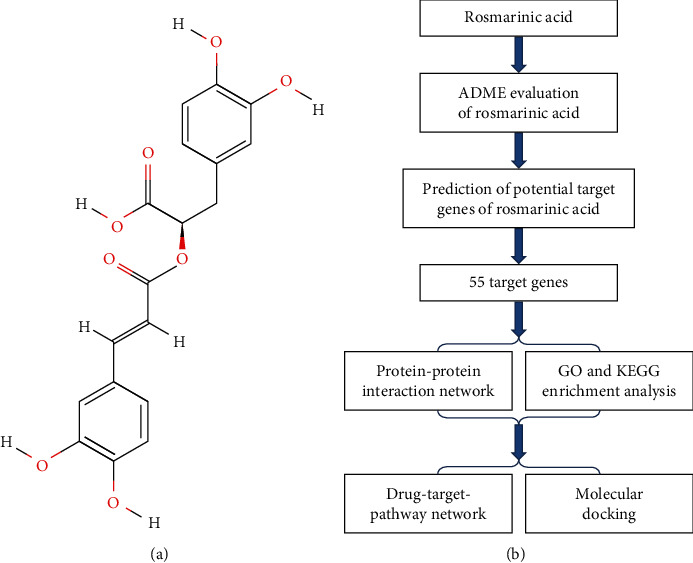
(a) Molecular structural formula of rosmarinic acid (PubChem CID: 5281792). (b) The flow of bioinformatics analysis of RosA includes target gene prediction, ADME evaluation, GO enrichment analysis, KEGG enrichment analysis, the construction of protein-protein interaction network, the construction of the drug-target-pathway network, and molecular docking.

In consequence, we systematically analyzed the pharmacological effects of RosA by using the method of network pharmacology. Firstly, the server of TCMSP was used to evaluate the biological characteristics of RosA. In addition, we predicted the potentially related target genes by chemical-gene interaction analysis. Furthermore, these potential target genes were used for GO enrichment analysis, KEGG enrichment analysis, and molecular docking. Finally, we systematically explained the potential targets and mechanism of RosA by constructing the pharmacological relationship network of RosA.  Figure 1(b) is the workflow of RosA gene prediction and analysis process.

## 2. Materials and Methods

### 2.1. Screening Chemical Structure from PubChem Database

PubChem database (https://pubchem.ncbi.nlm.nih.gov/) is an open and accessible database resource, which contains important information resources such as drug discovery and chemical biology research [13, 14]. We entered the keyword “rosmarinic acid” into the search box to retrieve the chemical formula (Figure 1(a)) and the CAS number of “RosA.”.

### 2.2. Evaluation of Pharmacokinetics by TCMSP

TCMSP Database and Analysis Platform (http://lsp.nwu.edu.cn/tcmsp.php) is one of the phytochemical databases. The database includes pharmacokinetic characteristics of natural compounds, such as oral bioavailability (OB), drug similarity (DL), blood-brain barrier (BBB), and water solubility [15]. Most of them can reflect the characteristics of drug absorption, distribution, metabolism, and excretion (ADME). Thus, in our study, the pharmacokinetic characteristics of RosA were searched and analyzed by the TCMSP database.

### 2.3. Screening of Gene Targets by the Comparative Toxicogenomics Database (CTD)

CTD (http://ctdbase.org/) is a database that aims to clarify the relationship between genes, drugs, and diseases. It provides key information about the interrelation between genes, gene interactions, disease, and chemical phenotype. The information in the database is selected from peer-reviewed scientific literature [16, 17]. The database was updated in 2019, which contains plenty of up-to-date information [18]. The target genes and phenotypes related to specific compounds can be retrieved by CTD and ranked according to the interaction. Therefore, we used this database to predict RosA's target genes. Genes with interaction ≥ 1 are selected as candidate target genes.

### 2.4. Construction and Analysis of Protein-Protein Interaction Network

The STRING 11.0 version (https://string-db.org/) is one of the online databases that can collect, score, and integrate all publicly available sources of information about protein-protein interactions. It can use computational predictions to supplement the existing information on protein-protein interactions. It achieves a comprehensive and objective global network, including direct (physical) and indirect (functional) interactions [19].

We uploaded 55 potential targets of RosA to the STRING database. The species was set as Homo sapiens and the minimum interaction score was 0.4 to build a protein interaction network. The results were imported into the Cytoscape3.7.2 version for visual analysis.

### 2.5. Gene Function and Pathway Enrichment Analysis

WebGestalt (http://www.webgestalt.org/option.php) is one of the popular software devices for functional and pathway enrichment analysis [20]. The GO is a comprehensive resource of the functions of genes and gene products containing molecular functions, biological pathways, and cellular components [21]. KEGG databases can give functional meanings to genes and genomes at the molecular levels and higher [22]. So, we input potential target genes into the WebGestalt server and use GO and KEGG databases for enrichment analysis.

### 2.6. Chemical Compound-Target-Pathway Network Construction

We have established a visual network through Cytoscape 3.7.2 version to further analyze and understand the complex relationships between RosA and targets and pathways.

### 2.7. Compound-Target Molecular Docking

Firstly, the chemical structure formula of RosA was downloaded from the CTD database and saved in mol2 format. Then, the 3D structures of target gene-associated proteins were downloaded from PDB data [23] (https://www.rcsb.org/) and saved in PDB format. The solvent molecules and ligands were removed by PyMOL software. AutoDock software was used to add hydrogenation, electron, and root and carry out other operations. And the formats of compounds and target proteins were converted to pdbqt format. During molecular docking, the protein structure was set as a rigid macromolecule, and the algorithm was Genetic Algorithm Parameters. For the group with the lowest binding energy of each protein in the results, PyMOL was used to draw 3D images, and one online tool (https://proteins.plus/) was used to generate 2D graphics.

## 3. Results

### 3.1. Molecular Formula and Pharmacokinetic Characteristics of RosA

Through the PubChem database, we obtained the chemical formula of RosA (Figure 1(a)). The CAS number is 20283-92-5. The database also provides some Depositor-Supplied Synonyms such as 537-15-5 (CAS number), and rosmarinic acid. At the same time, we obtained 12 essential ADME-related data of RosA through TCMSP (Table 1). As we can see from Table 1, MW is 360.34, DL is 0.35, OB is 1.38%, BBB is -1.24, AlogP is 2.69, and RBN is 7.

### 3.2. Prediction of Targets Gene of RosA

After that, we obtained the potential target genes related to RosA through the CTD database. We entered “rosmarinic acid” and used the default settings as a screening condition to search candidate genes. Then, we obtained 55 target genes related to RosA (Table 2). These target genes were used for further analysis.

### 3.3. Protein-Protein Interaction Network

**Figure 2:**
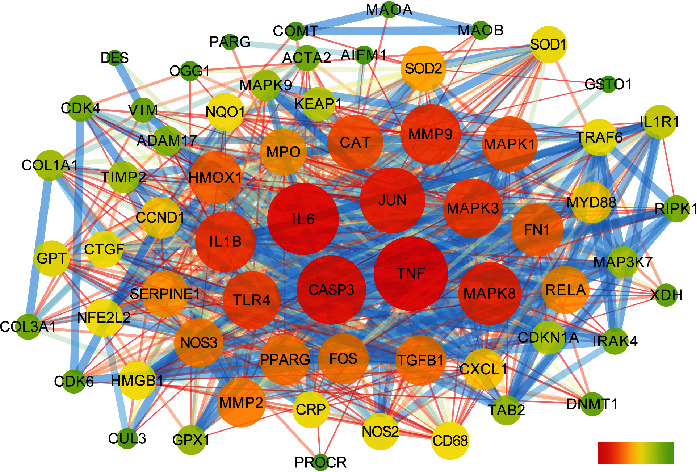
Protein interaction network of RosA target genes.

We imported the targets of RosA into the STRING database and selected organism as Homo sapiens to get the protein interaction network relationship. Then, we removed the free nodes and imported the results into Cytoscape 3.7.2 version for visualization (Figure 2). The size and color of the circle varied with the degree value. The width and color of edges varied with the combined score. There are 64 targets and 742 interactions in the picture. Calculated by the Network Analyzer plug-in in Cytoscape, the average degree value of the node is 23.19, the average Betweenness Centrality is 0.01, and the average Closeness Centrality is 0.61. Among them, there are 18 nodes whose degree value, Betweenness Centrality, and Closeness Centrality are all greater than the average. They may be the main targets for RosA to play a role. The detailed node parameters are shown in Table 3.

### 3.4. GO Enrichment Analysis

**Figure 3:**
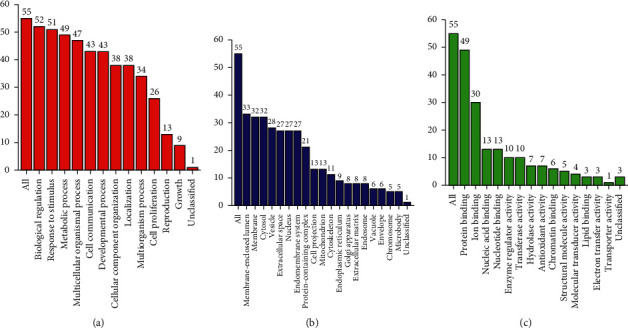
GO enrichment summary of the target genes. Biological process (BP), cellular component (CC), and molecular function (MF) are expressed by red (a), blue (b), and green (c), respectively. The height of the bar represents the number of target genes.

In order to further analyze the 55 selected genes, we carried out GO enrichment analysis of these candidate genes by WebGestalt tool. GO enrichment analysis showed that most of all potential genes were involved in biological process (BP), cellular component (CC), and molecular function (MF). BP enrichment mainly contained the following target genes: biological regulation (52/55), response to stimulus (51/55), metabolic process (49/55), multicellular organismal process (47/55), cell communication (43/55), developmental process (43/55), cellular component organization (38/55), localization (38/55), multiorganism process (34/55), and cell proliferation (26/55). CC enrichment was mainly involved in the following target genes: membrane-enclosed lumen (33/55), membrane (32/55), cytosol (32/55), vesicle (28/55), extracellular space (27/55), nucleus (27/55), endomembrane system (27/55), and protein-containing complex (21/55). MF enrichment was mainly engaged in the following target genes: protein binding (49/55) and ion binding (30/55). All the results are shown in Figure 3.

### 3.5. KEGG Enrichment Analysis

**Figure 4:**
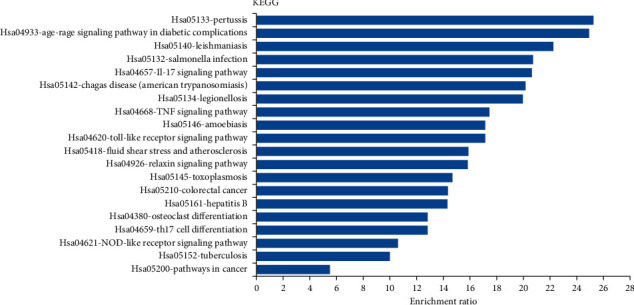
KEGG enrichment analysis of target genes.

At the same time, we carried out KEGG enrichment analysis of these candidate genes by WebGestalt tool. KEGG pathway enrichment analysis displayed that 55 potential target genes were enriched and 120 signal pathways were significantly related with the target genes (FDR ≤ 0.05). In Figure 4, we show the top 20 pathways with the highest enrichment ratio.

### 3.6. Network Analysis

**Figure 5:**
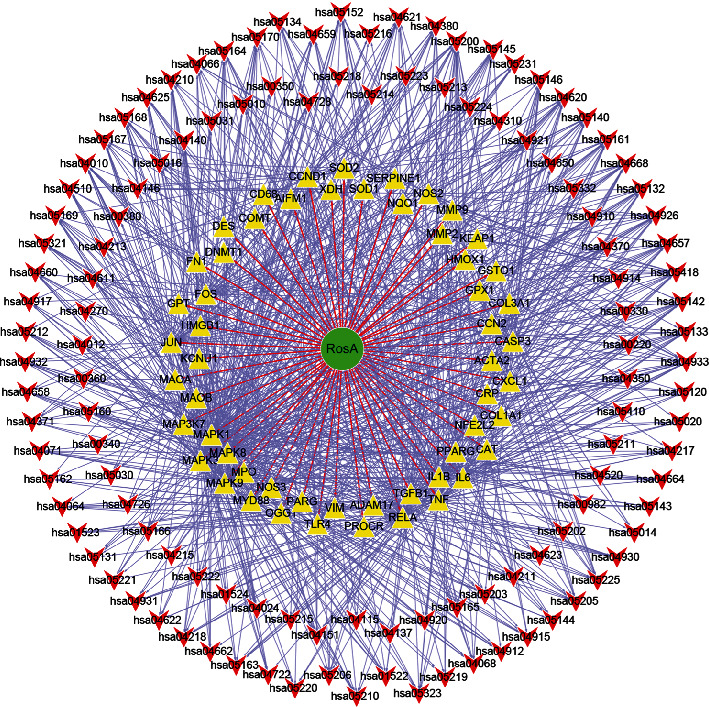
Compound (RosA)-target-pathway network.

In order to further clearly show the relationship between compound (RosA), targets, and pathway clearly, we constructed a drug-target-pathway network diagram by Cytoscape 3.7.2 version.  Figure 5 shows 176 nodes and 911 edges. Green circles represent compounds, yellow triangles correspond to targets, and red inverted triangles represent pathways.

### 3.7. Molecular Docking Results

**Figure 6:**
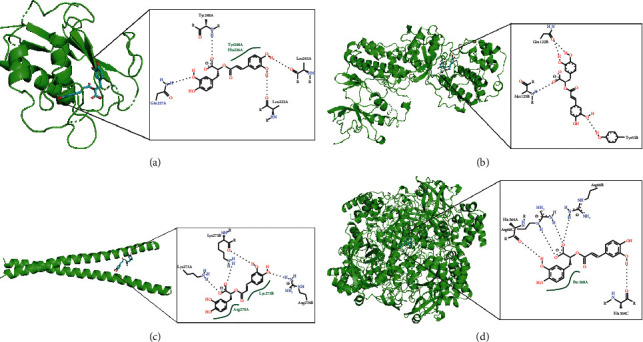
3D and 2D pictures of the four best docking results. (a) MMP9 and RosA; (b) MAPK3 and RosA; (c) JUN and RosA; (d) CAT and RosA.

It is generally believed that the lower the energy of the conformational stability of the ligand and the receptor, the greater the possibility of interaction. At present, there is no unified standard for target screening of active molecules. The active components with binding energy ≤−5.0 kJ/mol were selected as the basis for screening. The results of molecular docking showed that 13 of the selected target proteins had an affinity of less than −5.0 kJ/mol to RosA. The figure shows the best four docking results with the lowest binding energy. The results are shown in Table 4 and Figure 6.

## 4. Discussion

At present, the method of network pharmacology has been paid more and more attention during the process of drug development and utilization. This method can, firstly, evaluate, screen, and optimize some important characteristics of drugs, so as to speed up or simplify the process of drug discovery [11, 12]. Using this network analysis method, we not only obtained some important biological characteristics and some potentially related genes of RosA but also carried out GO and KEGG enrichment analysis.

Lipinski's Rule of Five is a rule of thumb. It is mainly used to evaluate whether a compound can be used as a drug or whether a compound with pharmacology or biological activity can become a human oral drug. Lipinski's rule of five mainly includes the following criteria: the molecular weight (MW) ≤500 g/mol; an octanol-water partition coefficient log P (Log P) ≤5; the donor of hydrogen bond (nitrogen or oxygen atoms with one or more hydrogen atoms) ≤5; the number of hydrogen bond receptors (nitrogen or oxygen atoms) ≤10 [24, 25]. The five rules are often referred to as guidelines for screening optimization of chemical libraries in the field of drug development [26]. At the same time, many studies have shown that the properties of compounds or drugs, such as molecular weight, lipophilicity, ionization, hydrogen bonding, polarity, and aromaticity, can affect their absorption, distribution, metabolism, excretion, and toxicity [27, 28]. It can be seen from Figure 1 and Table 1 that the pharmacokinetic characteristics of RosA are basically consistent with Lipinski's Rule of Five. At the same time, several studies suggested that RosA is metabolized by the gut microbiota into caffeic acid and its derivatives after ingestion [29–31]. And a higher metabolic rate was observed in the liver than in the intestine [32]. Then the caffeic acid produced by RosA is absorbed, combined, and methylated in tissues such as the digestive tract and liver to produce various metabolites [29].

DL is used to evaluate the “drug-like” degree of expected compounds. This helps to optimize pharmacokinetics and drug properties (such as solubility and chemical stability). The DL level of a compound is ≥0.18, which can be used as one of the screening criteria of the traditional Chinese herbs [33]. The DL value of RosA is 0.35. These all mean that RosA is one of the good candidates for drug development. However, the disadvantage of RosA is that its OB is relatively low. RBN are the number of bonds which allow free rotation around themselves. In particular, compounds which meet only the criteria of 10 or fewer rotatable bonds are predicted to have good oral bioavailability [34]. The RBN value of RosA is 7, which means that RosA may have a good oral bioavailability. What is worse, it is not easy to cross the BBB. However, some studies suggested that drug utilization can be improved by improving the extraction method of RosA [32] or by nanocarriers [35, 36]. But we still need to conduct further research and development on the practical application of RosA.

In the process of drug discovery, the search for target genes is a very important link. More and more molecules, compounds, and drugs have been found to have complex relationships with many genes and proteins [37–40]. At the same time, more and more online analysis tools have been developed in these studies. As shown in Table 2, 55 potential target genes related to RosA were identified by CTD database analysis. After that, we analyzed the 55 potential target genes through the STRING database. Degree value, Betweenness Centrality, and Closeness Centrality are all greater than the average of 18 nodes, most of which (such as TNF, IL6, CASP3, JUN, MAPK8, IL1B, MMP9, MAPK3, and TLR4) participate in the process of immune-inflammatory response. At the same time, in order to further explore the possible interaction between RosA and these targets, we made a molecular docking. When the conformational energy of the ligand binding to the receptor is stable, the lower the energy is, the greater the possibility of interaction is. Taking the binding energy ≤ −5.0 kJ·mol^−1^ as the screening criterion, we found that the binding energy of 13 of the 18 targets was less than −5.0 kJ·mol^−1^; that is, RosA may interact directly with these targets. So, they may be the key targets for the pharmacological action of RosA.

We did further GO analysis and pathway analysis of these potential genes through WebGestalt. According to the analysis of BP items, RosA is closely related to biological regulation, response to stimulus, metabolic process, multicellular organismal process, cell communication, developmental process, and so on. These results indicate that RosA may be related to anti-infection and antitumor. What is more, 9 of the first 20 enriched KEGG pathways are related to bacterial, viral, and parasite infections, such as pertussis, Chagas disease (American trypanosomiasis), salmonella infection, hepatitis B, amoebiasis, and toxoplasmosis. These results suggested that RosA may have antibacterial, antiviral, and anti-infective effects. Some of these pathways are related to inflammatory diseases and tumor diseases, for example, the AGE-RAGE signaling pathway in diabetic complications, colorectal cancer. Other studies suggested that RosA can inhibit the growth of cancer cells [41–43]. In addition, KEGG enrichment results also include IL-17 signaling pathway, TNF signaling pathway, Toll-like receptor signaling pathway, NOD-like receptor signaling pathway, Th17 cell differentiation, relaxing signaling pathway, and other immuno-inflammatory response-related pathways. Therefore, our study suggested that RosA may play a role by inhibiting excessive immune response, storm of inflammatory factors, and proliferation of tumor cells. Wei et al. suggested that RosA can reduce inflammation and atherosclerosis [44, 45]. Similarly, studies showed that RosA can also inhibit allergic inflammation in mice [46–48]. This is similar to our GO and KEGG analysis results. What is more, we made a drug-target network diagram, which further indicates that RosA may have a wide range of pharmacological activities.

## 5. Conclusions

Our studies showed that RosA has many pharmacological activities. At the same time, we analyzed the possible mechanism of action of RosA, which can be used to further develop safe and effective anti-inflammatory and anticancer drugs. Our work provides a new idea for the research, development, and clinical application of RosA. However, its shortcomings such as low oral availability and not easy to pass through the BBB also bring new challenges.

## Figures and Tables

**Table 1:** Pharmacological and molecular properties data of RosA.

Name	MW	AlogP	OB (%)	Caco-2	BBB	DL	RBN
RosA	360.34	2.69	1.38	−0.54	−1.24	0.35	7

MW, molecular weight; Caco-2, caco-2 permeability; OB, oral bioavailability; DL, drug-likeness; BBB, blood-brain barrier.

**Table 2:** Target genes of RosA from CTD database.

No.	Gene ID	Gene symbol	Description
1	10544	PROCR	Protein C receptor
2	6868	Adam17	Adam metallopeptidase domain 17
3	5970	RELA	RELA proto-oncogene, NF-kB subunit
4	7040	TGFB1	Transforming growth factor beta 1
5	7124	TNF	Tumor necrosis factor
6	3553	IL1B	Interleukin 1 beta
7	3569	IL6	Interleukin 6
8	5468	PPARG	Peroxisome proliferator-activated receptor gamma
9	847	CAT	Catalase
10	4780	NFE2L2	Nuclear factor, erythroid 2 like 2
11	1277	COL1A1	Collagen type I alpha 1 chain
12	1401	CRP	C-reactive protein
13	2919	CXCL1	C-X-C motif chemokine ligand 1
14	59	ACTA2	Actin alpha 2, smooth muscle
15	836	CASP3	Caspase 3
16	1490	CCN2	Cellular communication network factor 2
17	1281	COL3A1	Collagen type III alpha 1 chain
18	2876	GPX1	Glutathione peroxidase 1
19	9446	GSTO1	Glutathione S-transferase omega 1
20	3162	HMOX1	Heme oxygenase 1
21	9817	KEAP1	Kelch-like ECH-associated protein 1
22	4313	MMP2	Matrix metallopeptidase 2
23	4318	MMP9	Matrix metallopeptidase 9
24	4843	NOS2	Nitric oxide synthase 2
25	1728	NQO1	NAD(P)H-quinone dehydrogenase 1
26	5054	SERPINE1	Serpin family *E* member 1
27	6647	SOD1	Superoxide dismutase 1
28	6648	SOD2	Superoxide dismutase 2
29	7498	XDH	Xanthine dehydrogenase
30	9131	AIFM1	Apoptosis inducing factor mitochondria associated 1
31	595	CCND1	Cyclin D1
32	968	CD68	CD68 molecule
33	1312	COMT	catechol-O-methyltransferase
34	1674	DES	Desmin
35	1786	DNMT1	DNA methyltransferase 1
36	2335	FN1	Fibronectin 1
37	2353	FOS	Fos proto-oncogene, AP-1 transcription factor subunit
38	2875	GPT	Glutamic--pyruvic transaminase
39	3146	HMGB1	High mobility group box 1
40	3725	JUN	Jun proto-oncogene, AP-1 transcription factor subunit
41	157855	KCNU1	Potassium calcium-activated channel subfamily U member 1
42	4128	MAOA	Monoamine oxidase A
43	4129	MAOB	Monoamine oxidase B
44	6885	MAP3K7	Mitogen-activated protein kinase kinase kinase 7
45	5594	MAPK1	Mitogen-activated protein kinase 1
46	5595	MAPK3	Mitogen-activated protein kinase 3
47	5599	MAPK8	Mitogen-activated protein kinase 8
48	5601	MAPK9	Mitogen-activated protein kinase 9
49	4353	MPO	Myeloperoxidase
50	4615	MYD88	MYD88 innate immune signal transduction adaptor
51	4846	NOS3	Nitric oxide synthase 3
52	4968	OGG1	8-Oxoguanine DNA glycosylase
53	8505	PARG	Poly (ADP-ribose) glycohydrolase
54	7099	TLR4	Toll-like receptor 4
55	7431	VIM	Vimentin

**Table 3:** Key protein topological parameters of protein interaction network.

Name	Degree	Betweenness centrality	Closeness centrality
TNF	53	0.06924682	0.8630137
IL6	51	0.05915435	0.84
CASP3	49	0.08868983	0.81818182
JUN	46	0.03570932	0.7875
MAPK8	44	0.02681408	0.76829268
IL1B	42	0.01914187	0.75
MMP9	42	0.02381611	0.75
MAPK3	41	0.01732055	0.74117647
TLR4	40	0.01981721	0.73255814
CAT	38	0.07900048	0.71590909
MAPK1	38	0.0134504	0.71590909
HMOX1	36	0.01175721	0.7
FN1	36	0.01346746	0.7
FOS	34	0.02316259	0.68478261
TGFB1	34	0.03713298	0.68478261
SERPINE1	31	0.01151125	0.66315789
SOD2	30	0.03539503	0.64948454
NQO1	24	0.01251491	0.61165049

**Table 4:** Compound-target molecular docking binding energy.

No.	Targets	Compound	Binding energy (kJ/mol)
1	MMP9	RosA	−7.76
2	MAPK3		−7.71
3	JUN		−6.83
4	CAT		−6.68
5	SERPINE1		−6.61
6	MAPK8		−6.16
7	FN1		−6.09
8	FOS		−6.02
9	TGFB1		−5.87
10	TNF		−5.73
11	MAPK1		−5.57
12	TLR4		−5.5
13	IL1B		−5.31
14	NQO1		−4.76
15	CASP3		−4.73
16	SOD2		−4.72
17	HMOX1		−4.34
18	IL6		−3.55

## Data Availability

The data used to support the findings of this study are included within the article.
